# Human punishment is not primarily motivated by inequality

**DOI:** 10.1371/journal.pone.0171298

**Published:** 2017-02-10

**Authors:** Jesse Marczyk

**Affiliations:** Department of Psychology, New Mexico State University, Las Cruces, NM, United States of America; Tianjin University of Technology, CHINA

## Abstract

Previous theorizing about punishment has suggested that humans desire to punish inequality per se. However, the research supporting such an interpretation contains important methodological confounds. The main objective of the current experiment was to remove those confounds in order to test whether generating inequality per se is punished. Participants were recruited from an online market to take part in a wealth-alteration game with an ostensible second player. The participants were given an option to deduct from the other player’s payment as punishment for their behavior during the game. The results suggest that human punishment does not appear to be motivated by inequality per se, as inequality that was generated without inflicting costs on others was not reliably punished. Instead, punishment seems to respond primarily to the infliction of costs, with inequality only becoming relevant as a secondary input for punishment decisions. The theoretical significance of this finding is discussed in the context of its possible adaptive value.

## Introduction

Consider the following scenario: a tourist is vacationing in a poor, but scenic part of the world. During a trip through the local town the tourist is pickpocketed by a poor man, who quickly absconds with his wallet. While the tourist could chase down the pickpocket himself or report the theft to the local police, he decides against it, viewing the theft as permissible on the grounds that the thief was likely still worse off than he subsequently was. Upon his returning home, the tourist is assaulted by a local man simply for being better off than his assailant.

This scenario likely sounds strange, owing to the role that inequality is playing as a primary input for psychological punishment mechanisms. A punitive psychology that tolerates the infliction of costs if the perpetrator is worse off than the victim would likely be outcompeted by a psychology that disregarded inequality and instead punished when the benefits to doing so outweighed the costs. Similarly, punitive mechanisms that harmed others simply for being better off would appear needlessly costly, initiating conflicts where none need exist.

While this hypothetical scenario is admittedly exaggerated, there exists a body of literature that interprets humans as showing evidence of a psychology that uses a similar set of inputs to produce similar outputs. In a sentence, this literature claims that people are averse to inequality per se [1]; that is, at least some people desire to move in the direction of equitable outcomes with others, even if achieving that equality comes at a cost. This inequality-sensitive psychology is claimed to occasionally leads people to do things like inflict costs on others simply for being better off with no compensating benefit to the person inflicting those costs [2], refrain from punishing harm-doers that do not generate disadvantageous inequality [3–4], or discard or destroy resources rather than see them distributed unfairly [5–7]. There is even evidence that such inequality-sensitive mechanisms might exist in several nonhuman species as well [8–9]. While inequality has been demonstrated to play a role in human punishment decisions, the nature and extent of that role is incompletely understood. As the initial hypothetical example would suggest, it seems unlikely that people find inequality itself truly aversive, meaning that inequality aversion accounts require further clarification. The goal of the present research is to assess the role that inequality per se plays as an input for human punishment decisions once it has been separated out from other, confounding factors. This will allow us to see whether people are inequality averse in a general sense, as well as guide our understanding as to why and when we should expect them to be sensitive to matters of equitable outcomes.

Before examining some of the empirical literature on inequality aversion, it is worth considering what adaptive benefits might be conferred by psychological mechanisms that use inequality as an input governing punishment decisions. One possible function of this inequality aversion could be to level, or at least minimize, fitness differentials between individuals [3–4,10]. The general logic here is that, because selection operates at the level of *differential* reproductive success, inflicting costs on those who possess a proximate advantage over you would increase the punisher’s *relative* fitness in turn. Even if the individual inflicting costs on others is not absolutely better off, they are nevertheless gaining an eventual fitness advantage. Such a hypothesis is incomplete, however: specifically, while those *relative* benefits can be reaped by making others worse off than you, there is no need for the infliction of costs to cease once a leveling of fitness has been achieved. In a quick example, a male who monopolizes one mate is doing worse than a male who monopolizes two in terms of expected reproductive potential, and could improve his relative fitness if his rival only had one mate as well. However, the former male could do better still if he not only reduced his rival’s success, but also increased his own, poaching the second mate from the latter and creating inequality that favors himself.

A second theoretical account of inequality aversion can be arrived at through the consideration of association management and the value a disadvantaged individual presents as a potential associate to others. As noted by Tooby & Cosmides [11], individuals facing transient states of need can make better social investment targets than others, as they are more likely to place a higher marginal value on provided assistance than those not facing a need state, as well as more likely to provide a return on that investment in the future than those facing more chronic states of need. As such, altruism might be invested preferentially in transiently-needy individuals. Consistent with this prediction, across cultures, individuals viewed as needy and unlucky are viewed as better targets of altruism than individuals who are needy and lazy [12]. While there are important differences between altruistic systems and punitive ones [13], it is nevertheless possible that the outputs of those altruistic systems might interfere with those of the punitive ones, as it is difficult, perhaps impossible, to simultaneously benefit and punish the same target.

A similar analysis can be applied to the third-party level as well when considering social support: if third parties are less willing to engage in or support the punishment of needy individuals–or, in fact, if they preferentially support them in the hopes of building or strengthening their associations with them–then punishment of needy perpetrators becomes costlier, owing to the lack of moral support they would receive [14]. As such, inequality might serve as a cue for estimating the costs of punishment. If the estimated costs of engaging in punishment are perceived to be high, then we should expect to see a reduction of punishment behaviors in turn.

Importantly, these two perspectives make different predictions on the role of inequality in punishment decisions: the fitness-leveling perspective should predict that all forms of inequality would be punished, regardless of the context in which they arose: the benefits to making myself better off with respect to others should remain constant regardless of *why* those others are better off than me. The association management approach, by contrast, predicts that the inequality between two parties should factor into punishment decisions only when inequality is generated via behaviors that inflict costs on others, as it is those costs–not inequality per se–that generate the states of transient need to which our punitive psychology responds.

With that theoretical framing in mind, previous research on inequality aversion can be more profitably examined. As this literature is sizable, only four specific experiments will be discussed here. The first of these is a paper by Dawes et al [2]. In the experiment, participants played an economic game together in groups of four, with each player starting with a different initial payment. Each participant could use that payment to anonymously ‘buy’ positive or negative tokens for other players at the cost of one unit per token. When received, these tokens would subsequently either add or reduce three units from the payment of the player to whom they were assigned. As all tokens were purchased and assigned before the players were aware of what tokens they were receiving, there was no potential to assign them to others on the basis of reciprocity. Even preceding the presence of any personal losses or gains, subjects appeared willing to purchase these tokens for others, with negative tokens generally assigned to those with the most money and positive tokens assigned to those with the least. To the extent that buying negative tokens for others can be considered a form of punishment, some people appeared willing to punish others strictly on the grounds that they had more resources than average, not unlike the initial tourist example.

While these results could be taken as evidence for inequality aversion, another possible interpretation for them is that participants were buying tokens for others *in anticipation* of the behavior those others would take. The knowledge of the participants that others *will* be affecting their payoff could have led them to distribute benefits and punishments to others on the basis of predictions concerning future behavior, rather than on the basis of inequality per se. As this game resembles a public goods game–where the optimal strategy for maximizing the overall payment would be for each participant to contribute the maximum amount, resulting in three times the total initial pot–withholding altruism from others can be seen as inflicting a cost on them, especially when resources have been distributed in a random fashion, reducing the perception of strong rights to one’s good fortune. In other words, participants could have been using cues of inequality to estimate how likely each group member would be to deliver benefits to others, with those possessing greater wealth being deemed less likely to subsequently contribute. This effect could potentially even have been magnified further by the knowledge of the participants that future reciprocation of losses would be impossible (as all rounds were played with different group members).

Another intriguing set of findings was presented by Raihani & McAuliffe [3]. In order to examine the role inequality was playing in explicit punishment decisions, Raihani & McAuliffe [3] had participants play a taking game in pairs. Each participant began the experiment with a sum of money. One participant was given an option to take some amount of money from the other, after which the person who could have been taken from had the option to pay some of their money to punish (by reducing the payment of) the taker. The taker would begin with less money than their partner in two of the three conditions, and both the players would begin with an equal amount in the third; because of that initial income distribution, taking would, in the first condition, fail to alter the direction of initial inequality between the two, result in equal payments for both players in the second, or generate an inequality favoring the taker in the third. More punishment was subsequently observed in the condition where taking generated inequality that favored the taker; when that inequality was not generated–when the taking failed to alter the initial inequality or when it generated equality—participants failed to punish takers to a greater degree than non-takers. These results can also be considered consistent with the idea that it was disadvantageous inequality per se driving punishment; not the desire to punish losses.

A follow-up experiment employing a similar taking game that used a variable punishment method and an increased number of conditions [4] reported roles for both inequality and revenge in determining the extent of punishment, rather than just inequality. Further, as participants in this experiment had the option to deduct more than a single, set amount from the would-be takers, another interesting result emerged: the modal punishment amount was one in which the punisher reached equality with the taker. Though the punishers *could* have made their partner worse off than them, provided they were willing to suffer the costs of doing so, they often refrained.

While the results of these experiments [3–4] are intriguing, an important confound is present in both of these experiments: there was no way of punishing disadvantageous inequality that did not *also* involve punishing taking. No condition existed in which disadvantageous inequality could be generated without taking from the punisher. This leaves open the possibility that people were punishing in order to punish losses, but only did so appreciably in particular contexts; specifically, when inequality was also paired with losses. It is also worth noting that human punishment does not appear to seek equality more generally, as it did in Bone & Raihani [4]; when punishers could pay a fixed cost to inflict *any* amount of punishment on their target, such punishment modally generates advantageous inequality that favors the punisher [15].

The final experiment to consider by Blake et al [5] examined the development of inequality aversion across cultures and ages. Participants aged 4–15 played a distribution game in pairs. The experimenter would either present the children with a distribution of a food reward for them and their partner that was even (1–1) or one that favored one partner or the other (4–1 or 1–4, respectively). One child in the pair was responsible for rejecting or accepting the offer: if the child rejected it, both children got nothing; if the child accepted, both got the reward. Rejections thus made both members of the pair worse off (but equal), and one member of the pair was passive in the interaction, meaning that it was not the behavior of the passive partner being punished. Blake et al [5] report that children from every culture showed evidence of disadvantageous inequality aversion, frequently rejecting distributions that disfavored themselves.

In the context of inequality aversion, these findings could suggest that people are willing to avoid, or even punish, inequality per se, even if it makes them and others materially worse off, and even if those others did not even take part in the behavior that created the inequality. Again, however, these results are not so straightforward, as it is unclear what these rejections signify. Perhaps, rather than punishing inequality per se, rejections represent an attempt to alter the behavior of the experimenter doing the proposing, encouraging him to put a greater emphasis on the decider’s welfare [8]. Indeed, to the extent that neither child in the interaction has any strong claim to the lion’s share of the resources, uneven distributions of them could have been interpreted by the children as more of an infliction of harm, whereby the experimenter is failing to provide a requisite level of altruism or care for the child’s welfare, relative to others.

A consistent theme of this literature on inequality aversion, then, is that the welfare of one or more of the participants could have been, or actually was, traded off for the welfare of another in the service of creating various inequalities. In several instances, such tradeoffs could directly inflict costs [2–4], while in others, they could have represented failures to live up to social requirements [5]. This is an important confound, as it results in a lack of clarity concerning whether inequality per se is being targeted for punishment, or whether such punitive behavior is being driven by an aversion to suffering costs. Employing a methodology for removing this confound was the aim of the current research.

## The present research

The present research used a similar design to that found in Raihani & McAuliffe [3], but with a few important alterations. First, a free option for punishment was provided, as compared with the pay-to-punish methods frequently employed by past research. Participants were given the choice between either (a) deducting a set number of experimental points from another player’s payment for free or (b) not deducting. This modification was made in order to help disambiguate the desire to punish others (or avoid inequality) from the desire to avoid paying the costs of punishment; it is reasonable to suspect that people who would otherwise want to inflict costs on another would refrain from doing so if the costs of doing so were prohibitively high The free punishment option also allows us to more directly assess the outputs of particular preferences for inequality, rather than people’s abilities to instantiate those outputs in the world. For example, a weak individual may be unable to inflict punishment on a stronger one, even if the weaker one desires to do so. In much the same vein, researchers will often use ratings of physical attractiveness rather than actual dating behavior when assessing psychological attraction to others, as one might be unable to successfully mate with others he finds desirable.

The method through which inequality was generated was also split into three different conditions: inequality generated through taking, as before (where the participant suffers a loss while another player gains); inequality generated through losses to the participant alone (wealth destruction); and inequality generated through gains to the other player alone (wealth augmentation). If inequality per se–not losses–is generating punishment decisions, we should see a similar amount of punishment across all these conditions when disadvantageous inequality is generated; if, instead, the desire to punish losses is driving punishment decisions, we should see more punishment in the taking and wealth destruction condition, relative to the augmentation condition.

## Method

### Participants and procedure

All participants were recruited from Amazon’s Mechanical Turk website [16]. Participation was restricted to United States samples only, and written consent was obtained prior to participation. The final sample contained 309 (60% male) participants, distributed roughly evenly across all conditions. The average age of the participants was 29.78. The participants were told they would be assigned the role of player A or player B. Player A, the role to whom the participant was always assigned, would start the game with 100 points, which would be redeemed for money at the end of the experiment (at a rate of four points to one cent. All participants were paid the full twenty-five cents regardless of their condition).

From this point, the conditions differed: in the taking condition (N = 105; average age = 29.8; 60% male), the participants were told that player B started with 20, 60, or 100 points. In the first step of the experiment, player B had the option to take 20 points from the participant’s score and add it their own, or to not take anything. In all cases, the participant was told that player B selected the taking option. This taking would either fail to create inequality favoring the taker in the first condition (ending points for A and B would be 80/40, respectively), would create equality in the second (80/80), and would create disadvantageous inequality in the third (80/120).

In the wealth destruction and augmentation conditions, the participants were told that player B started with 60, 80, or 100 points. These point values were different from the taking condition owing to the fact that the relative point value between the players would be altered by 20, rather than 40 points in these latter two conditions. In the wealth destruction condition (N = 99; average age = 29.6; 63% male), the participant was told that player B had the option between deducting 20 points from the participant’s score or not deducting; in all cases, player B selected the deduction option. In the wealth augmentation condition (N = 105; average age = 29.9; 58% male), the participants were told that player B had the option of either adding 20 points to their own score or not making the addition; in all cases, player B opted to make the addition. This 20-point value was used in order to keep the absolute magnitude of gains to player B and losses to player A uniform with the taking condition. Again, the actions of player B would either fail to alter the state of the initial inequality (100/80 or 80/60 in the augmentation and destruction conditions, respectively), achieve equality (100/100 80/80), or create inequality favoring player B (100/120 or 80/100).

In all three conditions, following the actions of player B, the participant was given the choice between deducting 30 points from player B’s payment for free or not deducting and asked to rate the immorality of player B’s actions (from ‘not immoral at all’ to ‘extremely immoral’ on a 5-point scale). Following this decision, the participants were debriefed to the nature of the study and the deception regarding the existence of the second player, and paid the maximum amount of money. This deception was approved by the New Mexico State University review board and deemed necessary in order to collect a sufficient amount of data concerning pure wealth destruction decisions, which would otherwise be rare. Additionally, this deception allowed for the perception of losses to briefly exist without actually resulting in participants suffering any actual losses.

## Results

### Does the pattern of results obtained by Raihani & McAuliffe replicate?

Yes: when the taking generated disadvantageous inequality for the participants, they were more likely to engage in punishment (75%), relative to when the taking generated equality (47%) or when the participants were still better off than the other player (39%). When participants were being taken from, then, the presence of inequality favoring the taker appeared to make deduction decisions substantially more likely. A chi-square test confirmed that the difference between the first and second condition was significant, χ^2^ (1, *N* = 74) = 5.92, *p <* .05, whereas the difference between the second and third condition was not, χ^2^ (1, *N* = 69) = 0.52, ns.

### Does disadvantageous inequality per se increase deduction likelihood?

**Fig 1:**
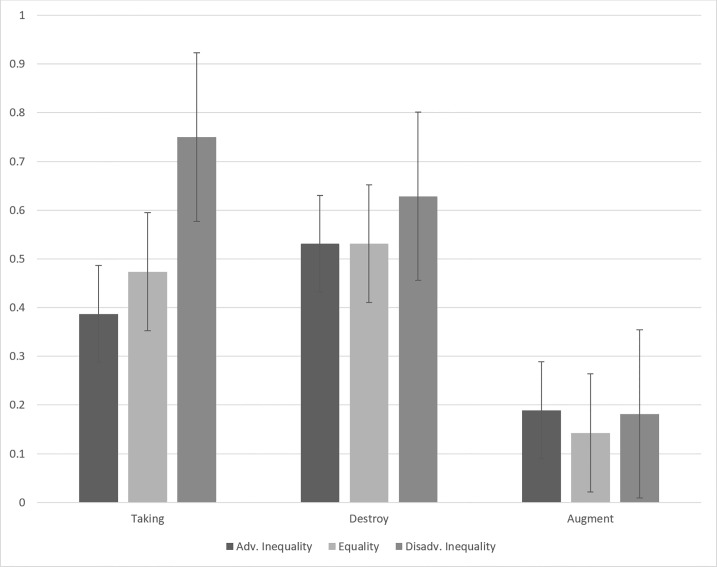
Percentage of participants deducting by condition.

No (see Fig 1). While participants experiencing losses alone without any corresponding benefit for the other player were nominally more likely to punish when the inequality favored the other player (63%), relative to when equality was created (51%) or the initial inequality did not change (53%), this difference was not significant, χ^2^ (2, *N* = 99) = 0.87, ns. Similarly, when the participant witnessed the other player experiencing gains alone without any corresponding cost to the participant, they were as likely to punish when the inequality favored the other player (18%), relative to when equality was created (14%) or the initial inequality did not change (19%), χ^2^ (2, *N* = 105) = 0.31, ns.

### Is generating inequality per se viewed as immoral?

No. A 3 (condition x 3 (inequality) ANOVA was conducted on the ratings of the immorality of player B’s actions. The ANOVA revealed a main effect of condition, *F* (2, 308) = 53.78, *p* < .001, ηρ^2^ = 0.25, no main effect of inequality, *F* (2, 308) = 2.52, ns, ηρ^2^ = 0.02, and no interaction, *F* (4, 308) = 1.64, ns, ηρ^2^ = 0.02. The mean immorality ratings (from 0 to 4) for each condition were *M* = 1.15 (*SD* = 1.08) for the taking condition, *M* = 1.75 (*SD* = 1.21) for the destruction condition, and *M* = 0.30 (*SD* = 0.80) for the augmentation condition. The difference between the taking and destruction conditions was significant, t (202) = 3.71, *p* < .001, as was the difference between taking and augmentation, t (208) = 6.46, *p* < .001

## Discussion

Previous research has claimed that human punishment behavior is motivated directly by inequality itself. However, that interpretation was drawn in the presence of important confounding factors, chief among which is that the possibility of experiencing losses (or interpreting the behavior or another as inflicting costs) was always present. When that confound was removed, punishment did not appear to be driven by an aversion to inequality per se; instead, punishment seemed to be driven primarily by the experience of losses.

In general, when the participants experienced losses, as compared to when they did not, they appeared more likely to deduct from the person who inflicted the losses, regardless of whether or not it generated disadvantageous inequality. If losses were not experienced, however, deductions were universally low, irrespective of the presence of inequality. Taken together, these results suggest that losses–not inequality–are the primary drivers of punishment decisions. While there is a role for inequality in modifying punishment decisions under specific circumstances, people do not appear to desire to punish inequality itself, as highlighted acutely by the results from the augmentation condition. This should be expected on the grounds that achieving equality with others does not, in and of itself, result in adaptive benefits, and may indeed carry costs at times, such as opposing mutually-beneficial courses of action [17]. Further, attempting to forcibly achieve equality with others would be expected to result in the initiation of frequent and unproductive conflicts. The costs of such conflicts might thus make the punisher better off with respect to the target of his punishment, but worse off with respect to many non-punishers in the population. A possible limitation of these results worth nothing is that while the present results suggest that inequality per se is not the main driver of punishment decisions—the focus of the experiment has been on inequality in the abstract, economic sense. It is possible that the role of inequality could be different in other domains, provided it can be examined independently of the perception of losses.

There are two primary ways to interpret the present findings, though both are broadly similar. The conservative interpretation is that inequality does not appear to impact punishment decisions in the absence of losses. In the augmentation condition, when disadvantageous inequality was intentionally generated (signaling a potentially unfair intention; [18]) *without inflicting costs* on the punisher, there was no increase in punishment, even nominally. The second, more speculative interpretation is that inequality does not appear to play a major role in punishment decisions until the welfare of the two parties is being traded off. While there was a nominal, but non-significant increase in punishment in the destruction condition, the overall gap between punishment in the equal and disadvantageous conditions was markedly smaller than in the taking group, suggesting some unique difference between how those conditions were assessed. For instance, the taking condition contains components of loss and altruism, whereas the destruction condition contains only loss. While the present results suggest that inequality per se is not the main driver of punishment decisions, the focus of the experiment has been on inequality in the abstract, economic sense. It is possible that the role of inequality could be different in other domains, provided it can be examined independently of the perception of losses.

The question remains as to why disadvantageous inequality seemed to potentiate punishment markedly in the taking, but not destruction or augmentation, condition. As initially speculated, two explanations are readily available and are not mutually exclusive. The first explanation is that needy individuals activate the altruism systems on the part of the second-party punisher (player A, in this case). As transiently-needy individuals are often better targets of altruistic investment, the outputs of the altruistic systems might interfere with the outputs of the punitive systems and suppress them, as one cannot target the same individual for punishment and investment at the same time. Some participants likely wished to benefit player B–at least initially–and letting B take from them was the only method by which that could be achieved. Importantly, this interpretation makes a solid prediction: when resources are earned, rather than randomly allocated, the role of inequality in punishment decisions should be mitigated. This prediction can be made on the grounds that individuals who are needy owing to their own behavior should present a lower association value than individuals who are needy owing to unfortunate luck: the difference between chronic and transient need states. Again, consistent with this prediction, previous research has found that altruistic attitudes towards individuals track the reasons for that need, with lazy targets receiving less support than unlucky, but motivated ones [12].

A second reason for this potentiation could reside in the estimated level of social support such punishment would receive from third parties [14]. In human societies, disputes often expand beyond dyadic conflicts, with third parties supporting one side, assisting in or defending against punishment. If third parties are willing to assist in the punishment of others, the social and physical costs of enacting punishment tend to decrease [14, 19–20]. It is possible that the present pattern of results could have obtained, in part, as the result of participants unconsciously estimating different costs of enacting punishment, contingent on expected third-party support (in spite of the fact that proximate costs remained constant). While no third parties were present in the current experiment, if our punitive psychology evolved in an environment where third parties often *did* join the disputes of others, we might expect that the cognitive mechanisms governing these decisions still behave under the assumption that third parties might become involved at some point. Considered in terms of the ecological topography of punishers [21], the presence of third parties in the vicinity of the punisher who support and/or are willing to engage in such punishment themselves might markedly decrease the proximate costs involved in punishment for every party involved. This could lead us to predict, then, that third parties should be more likely to endorse or support the punishing of those who (a) inflict costs on others or (b) take resources from another party and end up better off than their victim at greater rates than those who (c) simply benefit themselves or (d) take resources from another while ending up equally well off or worse off and (e) that the frequency of punishment behavior should vary contingent on their proximate costs, be those costs social or physical. In essence, third-party patterns of judgment should mirror the second-party patterns observed here [22].

This would, of course, raise the question as to why third-party support for punishment would sometimes–but not always–be magnified in the presence of inequality. One potential route for understanding why third parties care about inequality can be located in the same altruistic considerations mentioned initially, concerning how inequality might affect one’s association value to others [11,14]. An individual in a temporary state of need can prove to be a better target of limited social investment, relative to a satiated one, as the marginal values of resources channeled to the needy individual are larger. Accordingly, third parties might be more willing to support the transfer of resources from more to less needy individuals, as the expected social return on that support from the third party would be higher than directing their support towards already well-provisioned individuals. Indeed, this reasoning could also explain why the effects of inequality were not observed (or at least not as pronounced) in the destruction and augmentation conditions: destroyers were not receiving any comparable benefit, so supporting their harmful behavior would be unlikely to ingratiate the third party to the destroyer through helping him fulfill a need; similarly, supporting the punishment of individuals helping themselves at no cost to others would not help build social relationships through the fulfillment of a need on the part of the punishing party. As such it might only–or at least primarily–be when the welfare of two individuals is being traded off that the inequality between them enters into punishment decisions. Further, once inequality begins to enter into third-party punishment decisions, the local topography of punisher sentiments could be expected to change.

## Supporting information

S1 Fig Percentage of participants deducting by condition. (XLSX)
